# Omega-3 Fatty Acids Improve Functionality of High-Density Lipoprotein in Individuals With High Cardiovascular Risk: A Randomized, Parallel, Controlled and Double-Blind Clinical Trial

**DOI:** 10.3389/fnut.2021.767535

**Published:** 2022-02-23

**Authors:** Flávia De Conti Cartolano, Gabriela Duarte Dias, Sayuri Miyamoto, Nágila Raquel Teixeira Damasceno

**Affiliations:** ^1^Department of Nutrition, Faculty of Public Health, University of São Paulo (FSP-USP), São Paulo, Brazil; ^2^Laboratory of Modified Lipids, Department of Biochemistry, Chemistry Institute, University of São Paulo (IQ-USP), São Paulo, Brazil

**Keywords:** omega-3, high density lipoprotein, clinical trial, human, HDL subfractions

## Abstract

Omega-3 (ω-3) fatty acids have been extensively studied for primary and secondary prevention of cardiovascular health, but their ability to modulate HDL functionality remains unclear. The purpose of this study was to investigate the role of ω-3, rich in eicosapentaenoic (EPA) and docosahexaenoic (DHA), on HDL functionality. For that, 147 individuals with high cardiovascular risk were randomized in ω-3 (1 g of fish oil each - 370 mg of EPA and 230 mg of DHA, 3 times per day total EPA+DHA = 1,800 mg) or ω-6 groups (1 g of sunflower oil each - 760 mg of linoleic acid, 3 times per day; total linoleic acid = 2,280 mg). Fasting blood samples were collected at baseline time and after 8 weeks of follow-up and, and the lipid profile and glucose metabolism were evaluated from plasma. From HDL, the fatty acid profile, apolipoproteins (Apo AI, CII and CIII), paraoxonase-1 (PON1), cholesteryl ester transfer protein (CETP), subfractions and antioxidant activity were investigated. Omega-3 improved large HDL (HDL = 28.7%) and reduced small HDL (HDL10 = −10.6%) and the non-esterified fatty acids in HDL (NEFAs-HDL) level (−16.2%). A significant reduction in CETP activity was observed in the ω-3group (Δ ω-6 = 3.60 pmol/ul/h and Δ ω-3 = −1.99 pmol/ul/h; *p* = 0.044). The antioxidant capacity estimated by Lag time analysis did not change after the ω-3intervention. Changes in PON1 and Apo AI were inversely associated with increased incorporation of EPA (AOR = 0.446; IC = 0.200–0.994) and DHA (AOR = 0.351; IC = 0.150–0.821) in HDL, respectively. Cardioprotective profile obtained by pooled fatty acids analysis was related to a decrease in Apo CIII (r = −0.638; *p* = 0.002) and CETP (r = −0.341; *p* = 0.012) and an increase in Apo CII (r = 0.448; *p* = 0.042) and PON1 (r = 0.388; *p* = 0.003). In conclusion, omega-3 was effective in the reduction of cardiovascular risk associated with HDL functionality by size improvement and changes in its lipid, antioxidant and enzyme composition.

## Introduction

High-density lipoprotein (HDL) particle is probably the most heterogeneous and multifunctional lipoprotein in human. In addition to more than 110 different lipids and 70 proteins in its structure, HDL carries enzymes as paraoxonase (PON) and phospholipase A2 linked to lipoproteins (Lp-PLA2). Its complex composition prints to HDL an exclusive signature able to improve cardiovascular health. Currently, reverse cholesterol transport—the most studied function of HDL—divides the scenario of research with additional important functions, such as the endothelial stability, antioxidant effects, anti-thrombotic role and anti-inflammatory action (1).

Classically, the cholesterol in HDL has been associated to cardiovascular protection and reduced coronary heart events and mortality (2), however, the HDL effects appear to be largely affected by confounders. In the EPIC-Norfolk study, the protective action attributed to HDL disappeared after having included Apo B and triglycerides in regression models (3). In 2017, the controversy around the cardiovascular benefits of HDL grew when two important cohort studies including the Danish population (>350,000) showed an increased mortality in individuals with low and extremely high HDL-C levels. Based on that, extreme high HDL-C contributes to all-cause mortality in both men (74 mg/dL) and women (93 mg/dL) (4). Additionally, other components in HDL like apolipoproteins (Apo AI, Apo CII and Apo CIII), paraoxonase (PON1) and cholesteryl ester transfer protein (CETP) affect its structure, size and possibly its functionality (1, 2). Together, this background confirms that monitoring only cholesterol content in HDL is not enough to understand its complexity, functionality and anti-atherogenic role.

Historically, omega-3 (ω-3) fatty acid components present in diets have been used for prevention and management of cardiovascular risk factors and coronary heart diseases [for review, see (5–8)]. ω-3 and its metabolites, eicosapentaenoic acid (EPA) and docosahexaenoic acid (DHA), have been extensively investigated since the 1970's, when pioneer studies conducted by Bang and Dyerberg described direct association of total and fish fat with cardiovascular health (9–11). From that, several studies demonstrated that ω-3 is able to improve cardiovascular health by modulating different pathways linked to blood pressure control (12), glycemic metabolism (13), increase in expression and synthesis of lipoprotein lipase and, consequent reduction of triglycerides (14), improvement in lipid metabolism, decrease in low-density lipoprotein cholesterol (LDL-C) and increase in HDL-C (15), and decrease of very low-density lipoprotein (VLDL) (16). On the other hand, a systematic review and meta-analysis published in 2013 did not identify positive impact of ω-3 in cardiovascular disease (CVD) mortality (17). Recently, a Cochrane review concluded that ω-3 in diet and supplements were not associated with all-cause mortality (18). In addition to these controversies, a limited number of studies has investigated the potential connection of ω-3 and properties of HDL functionality (19, 20). These investigations are particularly attractive due to the successive unsuccess observed in clinical trials based on drugs focused on the cardioprotective role of HDL (21, 22). In the present study, we examined the impact of ω-3 fatty acids on the HDL functionality with the focus on the antioxidant capacity, composition and its subfraction distribution in individuals with high cardiovascular risk. The secondary outcomes of interest in this study were the impact of ω-3 on classical glycemic and lipid metabolism.

## Materials and Methods

### Design Study and Participants

This study was an 8-week randomized, controlled, parallel, double-blinded clinical trial. It was registered at the Brazilian website of clinical trials (www.ensaiosclinicos.gov.br) as RBR-2vfhfv. Individuals were recruited from the University Hospital of the University of São Paulo. A total of 188 individuals responded to advertisements to participate in the study, and 168 were assigned to treatments. Eligible subjects to this study were 30–74 years old with at least one cardiovascular risk factor (dyslipidemia, high blood pressure, diabetes, or smoking). During a prescreening assessment, participants completed a battery of questionnaires to obtain demographic and clinical profiles addressing sex, age, clinical information, family history of chronic diseases (father and mother), smoking status, blood pressure, and regular medication use. Exclusion criteria included cardiovascular events (assessed by clinical history and electrocardiogram—ECG), malnourishment, pregnant or lactating individuals, participants in other intervention protocols, patients with acute or chronic severe and uncontrolled diseases, users of illicit drugs, alcoholics, people allergic or intolerant to any component of the intervention and individuals with uncontrolled psychiatric disorders.

All participants were randomly allocated into either the omega-6 group (ω-6) or the omega-3 group (ω-3). Individuals received three capsules for daily consumption containing 1 g of fish oil each (370 mg of EPA and 230 mg of DHA – total of ω-3/per day = 3 g; 1,110 mg of EPA and 690 mg of DHA) administered to ω-3 group or 1 g of sunflower oil each (760 mg of linoleic acid) for ω-6 group. Capsules were purchased from the Health Industry (Indaiatuba, São Paulo, Brazil) that blinded both interventions in color, shape, size, packing, and smell. High performance liquid chromatography (HPLC) analysis by an independent laboratory confirmed the contents of fatty acids in the capsules. Adherence to interventions was monitored by the number of capsules returned at final follow-up time (T8). Patients were requested to undertake their regular physical activity and not to take any extra nutritional supplements during the 8-week trial. All patients completed a 2-d food record and 1 physical activity record (23) at the baseline of the study and at the end of the intervention. Both, 24 h recalls and physical activity questionnaire were applied by trained researchers and direct interviews. Energy and nutrient intakes were calculated by The Food Processor software (version 10.11.0) and intrapersonal variability was adjusted by The Multiple Source Method version 1.0.1 (DIfE, Nuthetal, German). After that, all nutrients were adjusted by energy. Anthropometric measures (weight, height, and waist circumference) and body composition (fat mass) were also evaluated by tetrapolar bioimpedance (Biodynamics 450®, TBW, Brazil). All procedures were obtained by direct evaluation and interview, except for 2nd food record, which was applied by phone by trained health professionals.

### Blood Collection and Plasma Analysis

Participants were seen in the early morning after a 12-h fast. At enrollment and 8-week, blood samples were collected in vacutainer tubes containing ethylenediaminetetraacetic acid (EDTA) (1.0 μg/mL). For serum, blood samples were collected in dry tubes, and all samples were maintained at 4 °C. Plasma and serum were separated by centrifugation at 1,500 g for 10 min at 4°C. Protease inhibitors containing aprotinin (10.0 μg/mL), benzamidine (10.0 μmol/L), and phenylmethylsulfonyl fluoride (PMSF) (5.0 μmol/L) and the antioxidant butylated hydroxytoluene (BHT) (100.0 μmol/L) were added. Plasma triacylglycerol (TAG), total cholesterol (TC), and HDL-C levels were measured using standard commercial kits (Labtest, Minas Gerais, Brazil). Low-density lipoprotein cholesterol (LDL-C) level was calculated using the Friedewald equation (1972). Apolipoproteins AI, B, CII, and CIII were determined by standard methods, using the autokit Apo AI, Apo B, Apo CII and Apo CII^®^, respectively (Randox Laboratories Ltd., UK). Plasma and HDL non-esterified fatty acid (NEFAs) concentrations were analyzed by the NEFA-HR(2)^®^ kit (Wako Chemicals GmbH, DE). Glucose and glycated hemoglobin (HbA1c) levels were measured by an enzymatic and colorimetric kit (Glucose PAP Liquiform^®^ and HbA1c Turbiquest, Labtest, Minas Gerais, Brazil). ELISA was used to measure serum insulin (Insulin Human Direct ELISA Kit^®^, Life Technologies, Grand Island, NY). Insulin resistance was calculated with the homeostasis model assessment-insulin resistance (HOMA-IR) as follows: fasting insulin concentration (μU/mL) x fasting glucose (mmol/L) / 22.5 (24). The PON1 activity in HDL was analyzed as proposed by Mackness et al. (25) and paraoxon-ethyl (Sigma, USA) as used as the substrate.

HDL subfractions were determined by the Lipoprint^®^ system (Quantimetrix Inc., Redondo Beach, California). This method is based on the separation and quantification of lipoprotein subfractions using non-denaturing polyacrylamide gel. The system uses a lipophilic dye, which binds to cholesterol in the lipoprotein particles. Ten subfractions were identified and then classified as HDL_LARGE_ (HDL1 to HDL3), HDL_INTERMEDIATE_ (HDL4 to HDL7), or HDL_SMALL_ (HDL8 to HDL10).

### Biochemistry Profile and Functionality of HDL

The antioxidant capacity of HDL was based on the lag time test described by Esterbauer et al. (26). Briefly, an LDL standard sample was diluted in PBS without EDTA to a final concentration of 0.083 mg/mL and distributed in flat-bottom plates containing 96 wells. Subsequently, HDL samples (200 mg/mL) precipitated by tungsten acid (2:1; v/v) from each of the patients' serum were added (Labtest, Minas Gerais, Brazil). Peroxidation was induced by CuSO_4_ (30 μmol/L), and absorbance was measured at 234 nm for 5 h. The oxidation resistance phase (lag time), the propagation phase of the conjugated dienes (indicated by the increase in absorbance), and the decomposition phase of these compounds (plateau) were observed. From the results, the lag time phase was calculated, as well as the maximum rate of lipid peroxidation (V_max_), the maximum production of conjugated dienes (DO_max_), the time for maximum production of conjugated dienes (T_max_) and the area under the curve generated (AUC).

For the HDL-fatty acid analysis, a solution formed by HDL (50 μL), methanol (1.75 mL), acetyl chloride (100 μL) and tridecane acid (50 μL) used as internal standard solution was mixed (30 s) in screw-capped glass tubes, then heated at 100 °C for 60 min and after that, cooled to room temperature. Hexane (1.5 mL) was added, and tubes were vortexed for 1 min. The samples were centrifuged at 1,500 x g for 2 min at 4 °C. The upper organic phase was collected, and this extraction procedure was repeated with hexane (0.75 mL) in order to optimize lipid extraction. The combined hexane solution was evaporated under nitrogen and the dry residue was then redissolved in hexane (100 μL). Samples were analyzed by gas chromatography with flame ionization detection (Trace 1310, Thermo Scientific) using a DB-FFAP column (15 m x 0.1 mm ID x 0.1 μm) (J and W Scientific from Agilent Technologies). Temperature program started at 150 °C with a 0.25 min hold, which was increased at 35°C/min to 200°C, 8°C/min to 250°C, followed by 4 min of isothermal period. Run time for a single sample was 14 min. Fatty acids in HDL were identified by direct comparison with a FAME standard mix (Supelco 37 Component FAME Mix; Sigma-Aldrich). Each fatty acid peak was integrated and then normalized by internal standard. All analyses were conducted in duplicate, and coefficients of intra- and inter-assay variance were 1– 15%. Results are expressed in percentage of all peaks integrated after to exclude internal standard.

### Statistical Analysis

The sample size was calculated based on the method previously proposed for clinical trials (Browner, Newman and Hulley 2008), using Student's *t* test to compare means of continuous variables, with a standardized magnitude of effect and specific bilateral α values of 0.05 and β of 0.10 (statistical power of 90%). For that, were considered biochemical parameters related to the outcomes: HDL-C, HDL size, HDL composition, and lag time. Based on this information, 60 individuals were the minimum required to be included in each intervention group.

All statistical analyses were performed by using SPSS^®^, version 20 (IBM Corporation). Statistical significance was set at a 2-sided level of *p* < 0.05. The distribution of the data was checked by using normal plots and histograms and by performing the Kolmogorov-Smirnov test (*p* > 0.05). Time effect was performed for each group using paired Student's *t* or Wilcoxon tests. Differences between groups were assessed after calculating the magnitude of the intervention effect (Δ = T8-T0) and its percentage variation (Δ% = (Δ/T0)^*^100) and were compared using unpaired Student's *t* or Mann-Whitney tests. Categorical variables were evaluated using Pearson' s chi-square test. To identify the effect of fatty acids on HDL functionality, univariate logistic regression analysis was performed using the EPA, DHA, or ω-6/ω-3 ratio as an independent factor. Afterwards, variables that showed correlations with EPA, DHA, or ω-6/ω-3 values (*p* < 0.20) have been included in the final model. Multiple adjusts for confounding variables such as sex, smoking, previous diseases, and use of medications, were tested. The adjusted odds ratio and 95% confidence interval (CI) were determined. In addition, the Principal Component Analysis (PCA) was applied to the fatty acid (Δ = T8-T0) incorporated into HDL to analyze the covariance structure of the variables and reveal a restricted number of sample standards. The original data were self-scaled, dividing the mean by the standard deviation, resulting in variables with the same weight. The recalculated data were then submitted to the PCA, and the results were presented when loading. The Eigenvalue method were used to extract the Principal Components (PC) and the Kaiser criteria was used to consider the sample adequacy measure. The PCA projected 7 significant PC (Eigenvalue > 1), which were correlated with the HDL functionality parameters. Based in the PCA method a summarized number of groups were obtained according fatty acids profile more atherogenic (PCA 1, 2, 4, 5 and 6) and less atherogenic (PCA 3 and 5).

## Result

**Figure 1:**
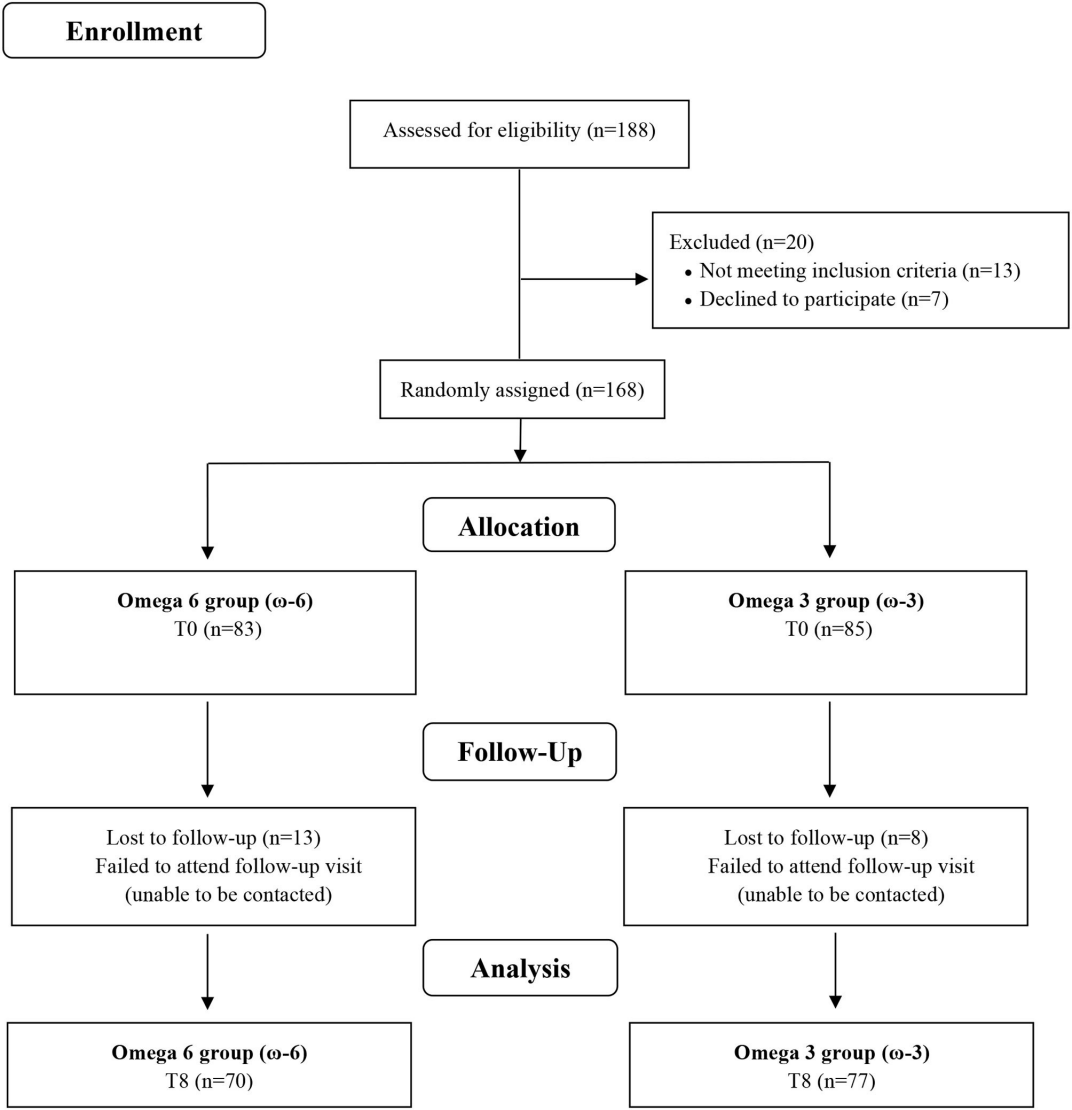
Flow chart of study.

Twenty of the 188 screened participants were excluded before being allocated to the intervention for not meeting the inclusion criteria (*n* = 13) or after having decided not to join the study (*n* = 7). The remaining 168 volunteers were enrolled and randomly assigned to ω-6 or ω-3 groups, where 13 and 8 participants dropped out during follow-up time, respectively (Figure 1). The baseline characteristics of the study participants are shown in (Table 1). There were no significant differences between the two intervention groups by age, sex, ethnicity, smoking, current disease, use of medicine, weight, BMI, WC, FM, and physical activity level. Overall, the compliance rate in this study was high: 83.8% of capsules were consumed throughout the study in the ω-6 group and 83.1% in the ω-3 group. No side effects were reported.

**Table 1:** Baseline characteristics of study participants who completed the randomized controlled trial^a^.

**Variables**	**ω-6 (*n* = 70)**	**ω-3 (*n* = 77)**	***p***
Age, y	51.7 (10.0)	51.6 (10.0)	0.950
Woman *(n, %)*	42 (60.0)	49 (63.6)	0.650
White ethnicity *(n, %)*	46 (65.7)	54 (70.1)	0.566
Smoking *(n, %)*	08 (11.4)	16 (20.8)	0.309
Current disease *(n, %)*	62 (88.6)	66 (85.7)	0.606
*Hypertension*	42 (60.0)	43 (55.8)	0.610
*Dyslipidemia*	37 (52.9)	38 (49.4)	0.671
*Diabetes mellitus*	15 (21.4)	12 (15.6)	0.361
*Others*	26 (37.1)	24 (31.2)	0.445
Use of medicine *(n, %)*	54 (77.1)	56 (72.7)	0.538
*Antihypertensives*	40 (57.1)	37 (48.1)	0.270
*Statins*	15 (21.4)	16 (20.8)	0.923
*Hypoglycemic agents*	16 (22.9)	13 (16.9)	0.363
*Fibrates*	02 (2.9)	02 (2.6)	0.420
*Others*	41 (58.6)	40 (51.9)	0.923
Weight, kg	83.8 (20.0)	82.4 (18.7)	0.658
BMI, kg/m^2^	31.4 (5.8)	30.7 (6.2)	0.475
WC ♂, cm	105.7 (12.8)	105.0 (12.6)	0.835
WC ♀, cm	101.1 (14.3)	97.0 (12.5)	0.146
FM ♂, %	24.4 (4.6)	23.6 (4.3)	0.525
FM ♀, %	44.1 (7.8)	43.9 (8.0)	0.880
Physical activity	6.89 (1.37)	7.13 (1.45)	0.951

a *Values are mean (SD), otherwise mentioned. P values were obtained using Pearson's Chi-square test for categorical variables and Student's t-test for continuous variables. BMI, body mass index; WC, waist circumference; FM, fat mass*.

Self-reported dietary intake at baseline (no differences between groups) and at the end of the study are given in (Table 2) and confirm that both groups had similar intake of calories and fat. Differences were found over time in the consumption of total fiber in both groups and a reduction in the consumption of carbohydrates in the ω-3 group. Regarding ω-3 intake, the intervention promoted an increase of 947% in the ω-3 group, while ω-6 was not related to a significant increase in the ω-6 group.

**Table 2:** Evaluation of the dietary intake of the study population, by time and intervention^a^.

**Variable**	**ω-6 (*****n*** **=** **70)**			**ω-3 (*****n*** **=** **77)**		
	**T0**	**T8**	***p***	**T0**	**T8**	***p***
Energy (kcal)	1,641.58 (385.86)	1,560.44 (413.37)	0.113	1,660.14 (481.94)	1,603.54 (417.45)	0.308
Proteins (g)	75.25 (20.09)	76.25 (20.42)	0.726	73.00 (24.70)	74.02 (20.42)	0.727
Carbohydrates (g)	217.39 (55.15)	207.44 (50.67)	0.157	224.73 (66.47)	207.60 (52.23)	**0.037**
Lipids (g)	54.81 (17.37)	50.58 (17.43)	0.084	53.98 (17.42)	54.19 (17.58)	0.932
Cholesterol (mg)	223.57 (99.83)	214.44 (79.13)	0.638	223.60 (121.51)	220.21 (64.57)	0.675
Total fiber (g)	16.31 (4.29)	15.03 (4.31)	**0.037**	16.15 (5.18)	14.44 (4.54)	**0.014**
Saturated fatty acids (g)	17.86 (6.03)	16.68 (6.78)	0.106	18.11 (6.59)	18.06 (6.68)	0.739
Monounsaturated fatty acids (g)	14.22 (5.90)	12.73 (4.24)	0.132	14.19 (5.62)	13.67 (4.04)	0.639
Polyunsaturated fatty acids (g)	6.53 (2.32)	5.90 (2.08)	0.051	6.03 (2.48)	6.06 (1.76)	0.931
*Linoleic acid (g)*	4.33 (1.79)	4.15 (1.56)	0.451	4.07 (1.95)	4.10 (1.25)	0.917
*Linolenic acid (g)*	0.53 (0.20)	0.51 (0.21)	0.404	0.50 (0.23)	0.51 (0.19)	0.690
*EPA (g)*	0.04 (0.08)	0.04 (0.04)	0.145	0.04 (0.05)	0.05 (0.05)	0.064
*DHA (g)*	0.10 (0.18)	0.12 (0.09)	0.423	0.10 (0.11)	0.14 (0.10)	0.220
*EPA + DHA (g)*	0.15 (0.26)	0.16 (0.13)	0.627	0.14 (0.16)	0.19 (0.15)	0.050
*EPA+DHA+supplement (g)*	0.15 (0.26)	0.16 (0.13)	0.627	0.14 (0.16)	1.99 (0.15)	**<0.001**
Trans fatty Acids (g)	1.51 (0.65)	1.56 (1.26)	0.735	1.52 (0.50)	1.60 (1.06)	0.493

a *Values are mean (SD). P-values were obtained using paired t or Wilcoxon tests. EPA, eicosapentaenoic acid; DHA, docosahexaenoic acid. P values in bold show significant differences (p < 0.05)*.

It was observed (Table 3) that the supplementation for 8 weeks with ω-3 fatty acid was effective in changing the EPA content (T0 = 0.74%, T8 = 2.40%; *p* < 0.001) and DHA (T0 = 1.96%, T8 = 3.27%; *p* < 0.001) in the HDL particles. Consequently, the total ω-3 fatty acids in HDL also increased significantly (T0 = 3.84%, T8 = 6.84%; *p* < 0.001). Conversely, a significant reduction in total ω-6 fatty acids was observed in this group, as a result of the decrease in γ-linolenic acid, dihomo-γ-linolenic acid and acid arachidonic. In the ω-6 group, there was a modest increase in total ω-6 fatty acids, linoleic acid and the ω-6/ω-3 ratio, as well as a small reduction in eicosadienic acid (C20:2n-6) and the EPA. These changes have had impacted positively differences between groups in favor of the ω-3 intervention, where total ω-6 was reduced and total ω-3 was increased. This profile was directly related to higher EPA and DHA in the HDL of individuals included in the ω-3 group associated to the simultaneous decrease in arachidonic and linoleic fatty acids. There was also a significant effect of the intervention on the ω-6/ω-3 ratio (ω-6 group = 4.06% and ω-3 group = −48.26%; *p* < 0.001).

**Table 3:** HDL fatty acid profile, by time and intervention^a^.

**Variables (%)**	**ω-6 (*****n*** **=** **70)**			**ω-3 (*****n*** **=** **77)**			*p* ^**^
	**T0**	**T8**	**Δ%**	**T0**	**T8**	**Δ%**	
Saturated fatty acids	37.26 (1.93)	37.20 (3.31)	−1.05	37.07 (2.14)	36.86 (1.69)	−0.48	0.344
*Myristic acid (C14:0)*	0.75 (0.16)	0.76 (0.19)	−0.60	0.75 (0.18)	0.73 (0.1)	−4.00	0.678
*Pentadecylic acid (C15:0)*	0.51 (0.20)	0.49 (0.19)	−6.07	0.47 (0.18)	0.48 (0.22)	−1.43	0.792
*Palmitic acid (C16:0)*	22.76 (1.90)	22.84 (3.03)	0.59	22.98 (2.17)	22.37 (1.93)^*^	−2.25	0.359
*Margaric acid (C17:0)*	0.53 (0.29)	0.49 (0.18)	−4.18	0.46 (0.12)	0.48 (0.16)	1.79	0.134
*Stearic acid (C18:0)*	9.97 (0.89)	9.96 (1.16)	−0.60	9.91 (1.04)	10.02 (1.15)	0.79	0.443
*Arachidic acid (C20:0)*	1.51 (0.70)	1.45 (0.67)	−9.03	1.35 (0.65)	1.46 (0.72)	10.42	0.464
*Heneicosanoic acid (C21:0)*	0.04 (0.07)	0.02 (0.05)^*^	−72.61	0.03 (0.06)	0.05 (0.10)	−50.97	0.452
*Behenic acid (C22:0)*	0.46 (0.14)	0.49 (0.23)	5.66	0.45 (0.19)	0.50 (0.26)	7.69	0.298
*Tricosylic acid (C23:0)*	0.25 (0.13)	0.23 (0.15)	−6.47	0.25 (0.18)	0.27 (0.22)	7.81	0.311
*Lignoceric acid (C24:0)*	0.48 (0.18)	0.48 (0.22)	5.00	0.43 (0.21)	0.51 (0.26)^*^	15.18	0.060
Monounsaturated fatty acids	23.25 (2.52)	22.59 (2.56)^*^	−3.23	22.98 (2.40)	21.95 (2.67)^*^	−3.72	0.312
*Myristoleic acid (C14:1n-5)*	0.20 (0.13)	0.19 (0.13)	−5.41	0.17 (0.14)	0.18 (0.14)	−6.36	0.594
*10-Pentadecenoic acid (C15:1n-5)*	0.18 (0.13)	0.17 (0.12)	−2.04	0.14 (0.13)	0.17 (0.12)	−0.49	0.889
*Palmitoleic acid (C16:1n-7)*	3.30 (0.98)	3.12 (0.79)	−7.74	3.23 (0.89)	3.12 (0.92)	−2.42	0.950
*10-Heptadecenoic acid (C17:1n-7)*	0.41 (0.13)	0.38 (0.11)	−1.22	0.37 (0.14)	0.36 (0.16)	−2.22	0.711
*Oleic acid (C18:1n-9)*	18.32 (2.32)	17.91 (2.28)	−2.34	18.23 (2.10)	17.14 (2.28)^*^	−5.75	**0.040**
*11-Eicosenoic acid (C20:1n-9)*	0.28 (0.22)	0.25 (0.18)	−14.84	0.21 (0.14)	0.25 (0.17)^*^	10.53	0.091
*Erucic acid (C22:1n-9)*	0.31 (0.22)	0.29 (0.24)	−2.93	0.30 (0.23)	0.30 (0.24)	1.44	0.897
*Nervonic acid (C24:1n-9)*	0.66 (0.26)	0.66 (0.27)	−1.33	0.70 (0.43)	0.78 (0.37)^*^	5.48	**0.046**
Omega 6	35.06 (3.52)	36.07 (4.27)^*^	2.65	35.73 (3.39)	33.98 (3.66)^*^	−6.04	**<0.001**
*Linoleic acid (C18:2n-6)*	23.44 (3.18)	24.55 (3.71)^*^	2.31	24.44 (3.70)	23.75 (3.92)	−3.96	**<0.001**
*γ-linolenic acid (C18:3n-6)*	0.67 (0.39)	0.64 (0.26)	−1.81	0.58 (0.27)	0.51 (0.25)^*^	−13.77	**0.027**
*Eicosadienoic acid (C20:2n-6)*	0.34 (0.14)	0.31 (0.12)^*^	−6.06	0.30 (0.13)	0.31 (0.14)	0.00	0.124
*Dihomo-γ-linolenic acid (C20:3n-6)*	2.14 (0.46)	2.12 (0.43)	−1.33	2.08 (0.52)	1.72 (0.39)^*^	−17.96	**<0.001**
*Arachidonic acid (C20:4n-6)*	8.22 (1.83)	8.18 (1.76)	3.48	8.11 (1.58)	7.42 (1.50)^*^	−9.93	**0.003**
*Docosadienoic acid (C22:2n-6)*	0.25 (0.25)	0.26 (0.31)	2.38	0.23 (0.23)	0.27 (0.40)	6.57	0.649
Omega 3	4.02 (0.90)	3.75 (0.85)	−1.07	3.84 (0.84)	6.84 (1.47)^*^	82.93	**<0.001**
*α-linolenic acid (C18:3n-3)*	1.07 (0.32)	1.05 (0.33)	1.24	0.96 (0.22)	0.99 (0.19)	4.94	0.139
*Eicosatrienoic acid (C20:3n-3)*	0.20 (0.16)	0.19 (0.15)	−4.71	0.18 (0.15)	0.19 (0.18)	−3.56	0.972
*Eicosapentaenoic acid (C20:5n-3)*	0.84 (0.36)	0.71 (0.25)^*^	−11.93	0.74 (0.31)	2.40 (0.92)^*^	220.26	**<0.001**
*Docosahexaenoic acid (C22:6n-3)*	1.90 (0.55)	1.80 (0.47)	−2.29	1.96 (0.59)	3.27 (0.68)^*^	68.31	**<0.001**
*EPA + DHA*	2.74 (0.78)	2.51 (0.64)^*^	−5.39	2.70 (0.75)	5.66 (1.43)^*^	114.23	**<0.001**
ω-6/ω-3 ratio	9.21 (2.46)	10.05 (2.25)^*^	4.06	9.77 (2.38)	5.27 (1.64)^*^	−48.26	**<0.001**

a *Values are mean (SD)*.

* *p < 0.05 vs. T0, p-values were obtained using paired t or Wilcoxon tests*.

** p < 0.05 between Δ%, p-values were obtained using paired t-Student or Mann-Whitney tests. EPA, eicosapentaenoic acid; DHA, docosahexaenoic acid; Δ%, [(T8-T0)/T0] * 100. P values in bold show significant differences (p < 0.05).

The effect of ω-3 on plasma and HDL biochemical parameters, according to time and intervention, is described in (Table 4). In both groups, a significant decrease in the values of TC, LDL-C, TG, and an increase in HDL-C after 8 weeks was noted, but no significant effect of the intervention was observed when the ω-6 and ω-3 groups were compared. HDL-C to Apo AI ratio increased significantly in both groups, but the isolated effect of fatty acids was not identified (*p* = 0.411; data not shown). Similarly, PON1 to HDL-C ratio did not show changes according to the fatty acid tested (*p* = 0.886; data not shown). However, the ω-3 intervention promoted significant reduction on plasma NEFAs (ω-6 group = 9.8%, ω-3 group = −6.0%; *p* = 0.018). Similarly, the ω-3 group showed a significant decrease in the concentration of HDL_NEFAs_ at 8-wk, and also when compared to the ω-6 group (ω-6 group = −3.4% and ω-3 group = −16.2%; *p* = 0.006). ω-3 was effective in decreasing the CETP activity (Δ ω-6 = 3.60 pmol/ul/h and Δ ω-3 = −1.99 pmol/ul/h; *p* = 0.044).

**Table 4:** Biochemical profile of the study population, by time and intervention^a^.

**Variable**	**ω-6 (*****n*** **=** **70)**			**ω-3 (*****n*** **=** **77)**			*p* ^**^
	**T0**	**T8**	**Δ%**	**T0**	**T8**	**Δ%**	
TC (mg/dL)	199.0 (43.0)	187.0 (47.0)^*^	−5.0	204.0 (37.0)	187.0 (38.0)^*^	−7.0	0.415
HDL-C (mg/dL)	35.0 (10.0)	40.0 (11.0)^*^	14.0	38.0 (10.0)	41.0 (11.0)^*^	12.0	0.805
LDL-C (mg/dL)	133.0 (40.0)	121.0 (44.0)^*^	−7.0	138.0 (36.0)	124.0 (36.0)^*^	−8.0	0.678
TG (mg/dL)	159.0 (90.0)	129.0 (69.0)^*^	−14.0	143.0 (81.0)	108.0 (54.0)^*^	−18.0	0.325
TG/HDL-C	5.2 (4.2)	3.8 (3.3)^*^	−19.4	4.4 (3.5)	2.9 (2.1)^*^	−23.3	0.401
non-HDL-C (mg/dL)	165.0 (43.0)	147.0 (47.0)^*^	−9.0	167.0 (37.0)	146.0 (37.0)^*^	−11.0	0.579
Apo AI (mg/dL)	127.1 (25.3)	130.2 (22.4)	4.7	130.1 (25.4)	134.6 (33.2)	6.1	0.783
Apo B (mg/dL)	103.3 (24.9)	105.6 (26.6)	3.5	103.2 (24.8)	106.7 (37.1)	6.0	0.822
LDL-C/Apo B	1.3 (0.2)	1.1 (0.2)^*^	−9.9	1.4 (0.3)	1.2 (0.3)^*^	−10.4	0.910
NEFAs (mEq/dL)	0.59 (0.27)	0.64 (0.26)	9.8	0.68 (0.32)	0.63 (0.31)	−6.0	**0.018**
Glycemia (mg/dL)	108.0 (35.0)	107.0 (37.0)^*^	−1.0	101.0 (26.0)	103.0 (33.0)	2.0	0.428
Insulin (μIU/mL)	17.6 (7.5)	18.0 (7.9)	0.0	18.7 (8.5)	18.4 (8.4)	−0.5	0.876
HOMA-IR	4.7 (2.6)	4.8 (2.9)	1.7	4.5 (2.1)	4.5 (2.0)	−0.6	0.836
HbA1c (%)	5.1 (0.6)	5.1 (0.6)	0.0	5.0 (0.5)	5.0 (0.5)	0.0	0.447
PON1 (nmol min-1. ml-1)	59.0 (32.5)	59.2 (32.6)	1.5	54.7 (29.7)	55.7 (31.8)	5.2	0.498
CETP (pmol/ul/h)	50.3 (19.8)	53.7 (21.6)	14.9	49.0 (16.7)	49.1 (17.7)	4.6	**0.044**
HDL_ApoAI_ (mg/dL)	127,1 (25,3)	130,2 (22,4)	4,7	130,1 (25,4)	134,6 (33,2)	6,1	0,783
HDL_NEFAs_ (mEq/dL)	0.29 (0.15)	0.29 (0.14)	−3.4	0.32 (0.14)	0.25 (0.12)^*^	−16.2	**0.006**
HDL_ApoCII_ (mg/dL)	3.1 (1.5)	3.2 (1.8)	−8.4	3.0 (1.0)	2.7 (1.0)	−10.0	0.549
HDL_ApoCIII_ (mg/dL)	5.2 (2.3)	5.6 (2.8)	−8.7	5.1 (1.7)	4.6 (2.2)	−16.9	0.322

a *Values are mean (SD)*.

* *p < 0.05 vs. T0, p-values were obtained using paired t or Wilcoxon tests*.

** p < 0.05 between Δ%, p-values were obtained using paired t-Student or Mann-Whitney tests. TC, total cholesterol; HDL-C, high-density lipoprotein cholesterol; LDL-C, low-density lipoprotein cholesterol; TG, triglycerides; Apo, apolipoprotein; NEFAs, non-esterified fatty acids, HOMA-IR; homeostasis model assessment of insulin resistance; HbA1c, glycated hemoglobin; PON1, paraoxonase-1; CETP, cholesteryl ester transfer protein; Δ%, [(T8-T0)/T0] * 100. P values in bold show significant differences (p < 0.05).

**Figure 2:**
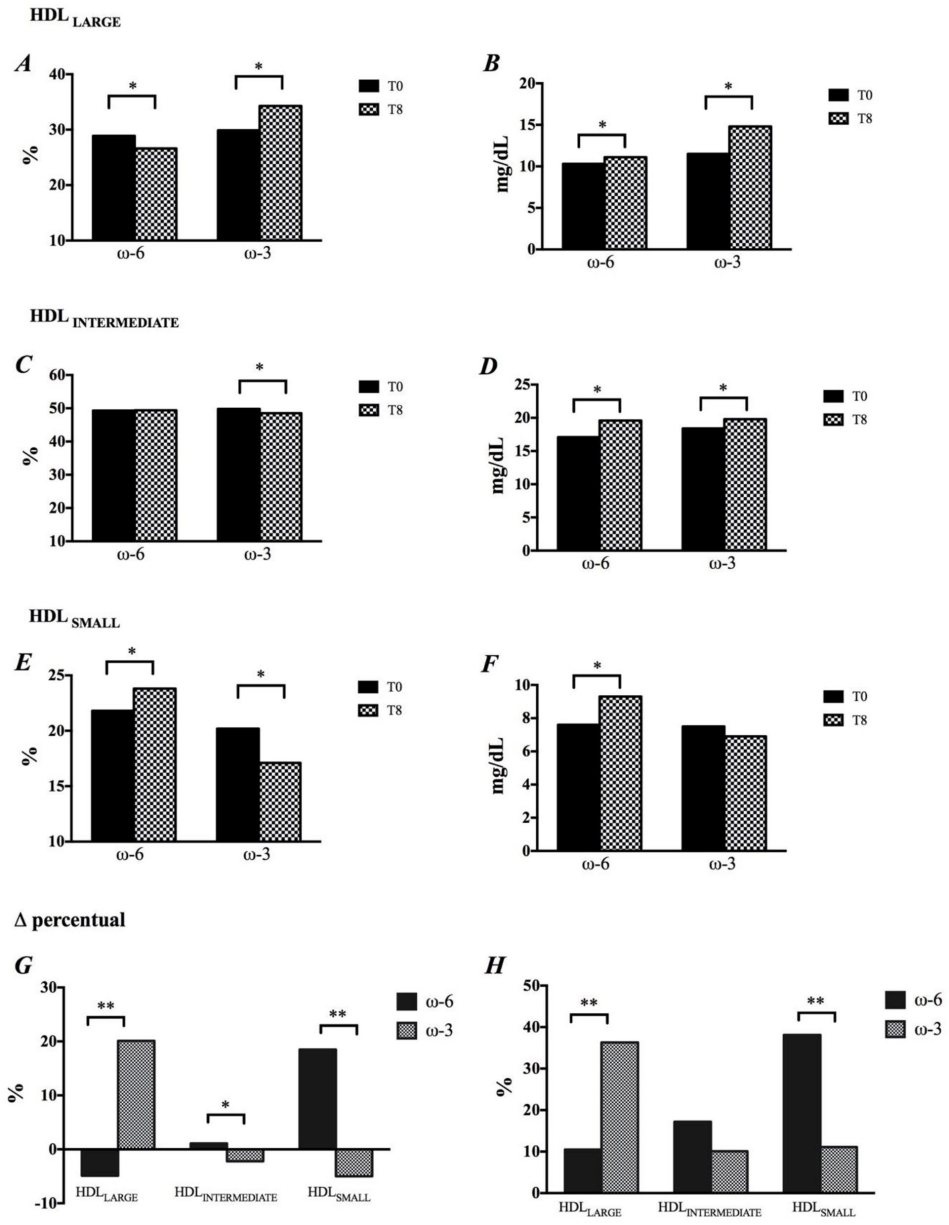
Modification in HDL size, by time and intervention. **(A,B)** HDLLARGE, **(C,D)** HDLINTERMEDIATE, **(E,F)** HDLSMALL, **(G)** Changes in HDL subfractions are shown in percentage, and **(H)** Concentration. *p < 0.05, *p*-values were obtained using paired *t* or Wilcoxon tests. **p < 0.05, *p*-values were obtained using paired *t-Student* or Mann-Whitney tests.

The intervention rich in EPA and DHA resulted in a significant increase in the percentage of HDL 1 (28.7%), HDL 2 (19.3%) and HDL 3 (19.8%) particles (Supplementary Table 1) and, consequently, in the percentage of HDL_LARGE_ (Figures 2A,G). There was a reduction in the percentage of HDL_SMALL_ (Figures 2E,G) as a result of the decrease in HDL 8 (-2.5%), HDL 9 (-6.7%) and HDL 10 (-10.6%) subfractions (Supplementary Table 1) The ω-3 promoted significant decrease in HDL_INTERMEDIATE_ (Figure 2C). The impact of ω-3 on HDL subfractions was reinforced after adjustment of the percentage of subfractions by the cholesterol concentration in this lipoprotein (Figures 2B,D,F,H, and Supplementary Table 1). Nevertheless, there were no changes in the HDL antioxidant capacity in function of the intervention (Supplementary Figure 2).

**Figure 3:**
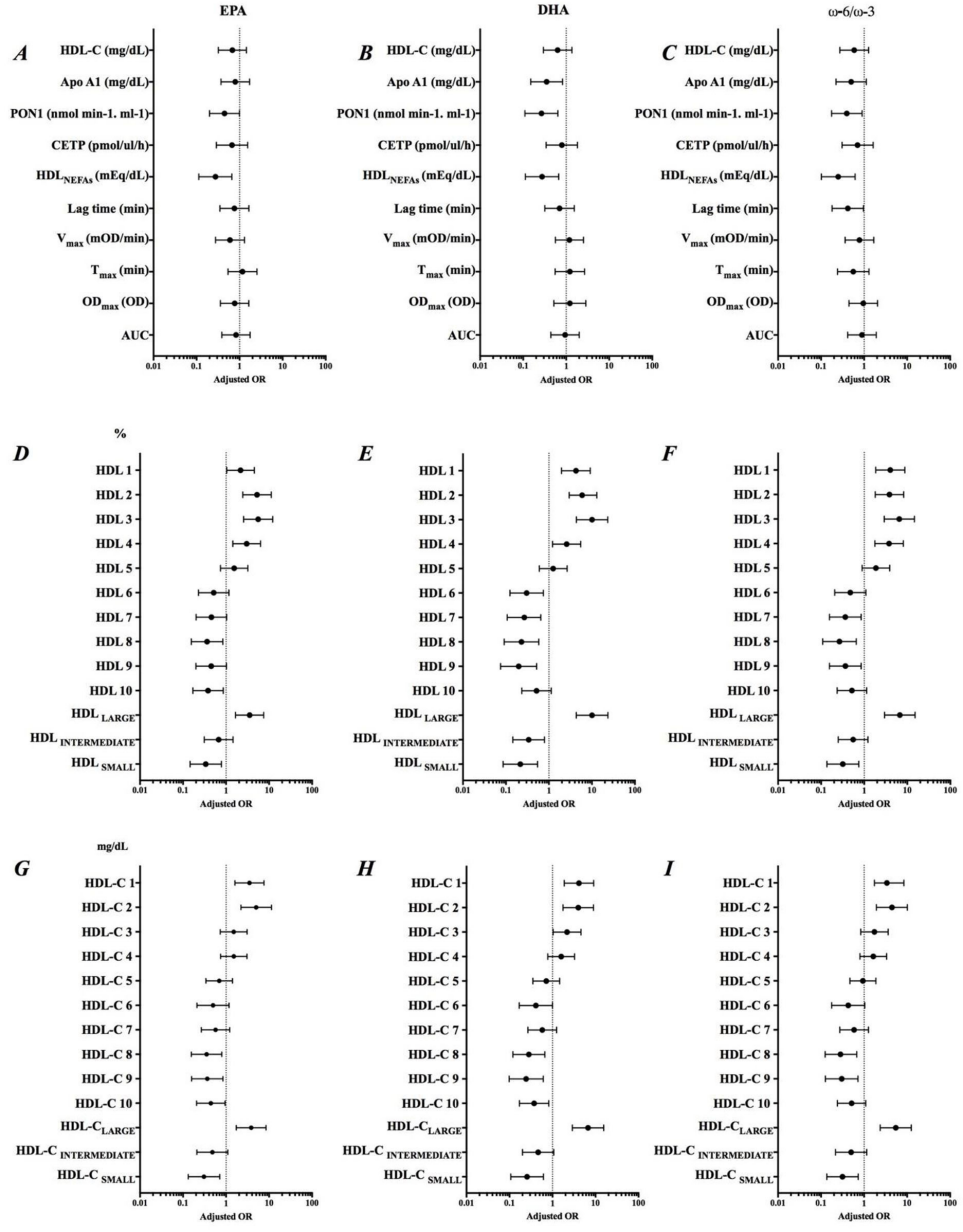
Adjusted odds ratio (AOR) of **Δ** EPA, **Δ** DHA and **Δ** ω-6/ω-3 ratio and variation of parameters related to HDL functionality. Adjustment by sex, smoking, previous diseases and use of medications. **(A–C)** Role of EPA, DHA and ω-6/ω-3 on HDL composition, **(D–F)** Percentage of HDL subfractions, and **(G–I)** Levels of HDL subfractions.

Adjusted odds ratio of EPA, DHA, and ω-6/ω-3 ratio and parameters related to HDL functionality are shown in (Figure 3). The increase in EPA content in HDL was associated with a lower chance of an increase in the PON1 activity (AOR = 0.446; IC = 0.200-0.994), HDL_NEFAs_ (AOR = 0.275; IC = 0.113-0.660), and HDL_SMALL_ (%) (AOR = 0.306; IC = 0.132-0.710) and HDL-C_SMALL_ (mg/dL) (AOR = 0.337; IC = 0.146-0.782). EPA was associated with approximately 3.5 more chances of increasing HDL_LARGE_ (%) (AOR = 3.522; IC = 1.652-7.507) and HDL-C_LARGE_ (mg/dL) (AOR = 3.826; IC = 1.725-8.488), while DHA was significantly associated with decreased Apo AI (AOR = 0.351; IC = 0.150-0.821). The decrease in the ω-6/ω-3 ratio reduced the chances of an increase in the resistance time due to oxidation of HDL by almost 60% (AOR = 0.418; IC = 0.180-0.969). Summarizing, the increased incorporation of ω-3 in HDL was associated with improvement in parameters linked to RCT, but not in the antioxidant components.

Results from principal component (PC) analysis projected seven fatty acid groups. Results were grouped in cardioprotective components (PC1, PC2, PC4, PC5, and PC6) that showed high content of unsaturated fatty acids, and atherogenic components (PC3 and PC7) in which the contribution of the saturated fatty acids and arachidonic acid was increased. Together, these seven components explained 78.16% of the fatty acids profile in HDL after intervention (Supplementary Table 3). Table 5 shows the correlations between the PC formed by fatty acids incorporated into HDL after intervention and the HDL functionality parameters. It was observed that PC2 showed a positive correlation with HDL_ApoCII_ (r = 0.448; *p* = 0.042) and PON1 (r = 0.388; *p* = 0.003), while PC5 presented a negative correlation with HDL_ApoCIII_ (r = −0.638; *p* = 0.002) and CETP (r = −0.341; *p* = 0.012). The PC6 correlated positively with the variation in the HDL_LARGE_ (%) (r = 0.420; *p* = 0.001) and HDL-C_LARGE_ (mg/dL) (r = 0.270; *p* = 0.042) and negatively with the HDL_SMALL_ (%) (r = −0.310; *p* = 0.019) and HDL-C_SMALL_ (mg/dL) (r = −0.334; *p* = 0.011). CP7 was positively correlated with HDL_SMALL_ (%) (r = 0.312; *p* = 0.018) and HDL_SMALL_ (mg/dL) (r = 0.293; *p* = 0.027). PC3 was negatively correlated with the antioxidant capacity of HDL (r = 0.275; *p* = 0.038).

**Table 5:** Correlation between principal components (PC) and parameters of HDL functionality^a^.

**Principal components**		**Δ HDL_**ApoCII**_**	**Δ HDL_**ApoCIII**_**	**Δ PON1**	**Δ CETP**	**Δ HDL_**LARGE**_**	**Δ HDL_**SMALL**_**	**Δ HDL-C_**LARGE**_**	**Δ HDL-C_**SMALL**_**	**Δ** **Conjugated** **dienes** **(AUC)**
PC1 (15.04%)^*^	r	−0.278	−0.208	0.154	−0.109	0.217	−0.248	0.203	−0.232	0.022
	p	0.223	0.366	0.254	0.439	0.105	0.063	0.131	0.082	0.873
PC2 (13.19%)^*^	r	**0.448**	0.086	**0.388**	−0.046	0.027	−0.058	−0.015	−0.078	0.073
	p	**0.042**	0.712	**0.003**	0.744	0.840	0.666	0.911	0.566	0.590
PC3 (12.45%)^**^	r	0.260	0.284	0.214	−0.004	0.098	−0.162	0.063	−0.074	**−0.275**
	p	0.256	0.211	0.111	0.976	0.467	0.228	0.639	0.583	**0.038**
PC4 (10.35%)^*^	r	0.161	0.368	0.032	0.009	−0.138	0.143	−0.138	0.107	−0.141
	p	0.486	0.101	0.814	0.947	0.307	0.288	0.306	0.427	0.295
PC5 (9.42%)^*^	r	−0.183	**−0.638**	−0.185	**−0.341**	0.147	−0.092	0.101	−0.169	−0.074
	p	0.427	**0.002**	0.167	**0.012**	0.276	0.497	0.453	0.209	0.585
PC6 (9.14%) ^*^	r	0.048	0.168	0.122	−0.031	**0.420**	**−0.310**	**0.270**	**−0.334**	−0.037
	p	0.836	0.468	0.365	0.823	**0.001**	**0.019**	**0.042**	**0.011**	0.786
PC7 (8.59%)^**^	r	−0.197	−0.239	0.099	0.052	−0.110	**0.312**	−0.125	**0.293**	0.156
	p	0.391	0.297	0.463	0.710	0.415	**0.018**	0.353	**0.027**	0.248

a *Values are correlation coefficients (r). p-values were obtained using Pearson or Spearman tests. PC, principal component; HDL, high-density lipoprotein; Apo, apolipoprotein; PON1, paraoxonase-1; CETP, cholesteryl ester transfer protein; AUC, area under curve; Δ, delta T8-T0*.

* *cardioprotective profile*;

** *atherogenic profile*.

## Discussion

In the last decades, the cardioprotective role of HDL has been extensively investigated and directly associated with its ability to transport cholesterol, making TRC the most important function of this lipoprotein (26). The present study extends the cardioprotective role of HDL by showing that ω-3 supplementation, rich in EPA and DHA, promotes modifications in several parameters of the HDL functionality, where we highlighted the increase in HDL-C concentration and large HDL particles, followed by the reduction of small subfractions and concentration of HDL-associated NEFAs, without, however, changing the antioxidant capacity of the particle. Our results highlight that the relationship between HDL and cardiovascular health involves physicochemical aspects that go beyond cholesterol content and thus our results contribute to the understanding of some controversies about HDL (27, 28).

In the Brazilian population, the ELSA study showed that individuals with higher concentrations of HDL-C (>90 mg/dL) showed less thickness of the intimal layer of the arteries (29); however, Madsen et al. (4) described that men and women with extremely high HDL-C showed high all-cause mortality (4). Here, an 8-week supplementation with ω-3 led to a significant 12% increase in HDL-C content, mainly due to an increase of 36% in the cholesterol associated with the larger particles, while the smaller particles showed an increase of 11%. Likewise, Mori et al. (30) also described that the increase in HDL-C observed in obese men who received 4 g of DHA for 6 weeks was due to a 29% increase in the cholesterol associated with the large HDL particles (30).

It has been suggested that HDL-C is more strongly influenced by DHA-enriched fish oils than those enriched with EPA (31). Our results show the role of ω-3 through the positive correlations between HDL-C and the changes in EPA (r = 0.276; *p* = 0.001) and DHA (r = 0.326; *p* < 0.001) and confirmed by association between these fatty acids and large HDL particles, where incorporation of DHA increased the likelihood by 6.7-fold to observe an increase in cholesterol in large HDL, while EPA was associated 3.8 times. It is noteworthy that, although the ω-6 group also showed an increase in HDL-C, this was mainly due to the increase in cholesterol associated with the small particles (38%), leading to only a modest increase of the larger particles (10%). Maki et al., in a double-blind, randomized, 6-week follow-up study including subjects with isolated hypercholesterolemia who were supplemented with 3.6 g of EPA+DHA, described a 1.5% increase in HDL-C concentration over time and a 3.3% increase when compared to placebo group, a more modest result than that observed in the present study (32).

Small, dense HDL particles have more antioxidant and anti-inflammatory properties than large particles, reducing LDL oxidation in the subendothelial layer (KONTUSH and CHAPAMAN, 2010) (33). More recently, Du et al. (34) showed that smaller HDL particles are more efficient in cholesterol efflux in macrophages, via ABCA1-like receptors (34). The present study showed that incorporation of EPA and DHA into the HDL particle promotes significant changes in the distribution of the subfractions of this lipoprotein, expressed as percentage, as well as after adjustment for the cholesterol content in each subfraction. This profile translated into a 20% increase in the larger HDL in the ω-3 group, raising the likelihood of the incorporation of EPA and DHA in increasing 3.5-fold and 10.0-fold, respectively, the chances of HDL becoming larger.

Although ω-3 supplementation, rich in EPA and DHA, plays a role in several steps of lipid metabolism, the action of ω-3 in lowering TG is certainly the most studied one (35). There is strong evidence that EPA and DHA consumption decreases TG levels in a dose-dependent manner, by reducing synthesis and increasing the catabolism of VLDL (36). Doses of EPA+DHA between 2 and 4 g can lead to 12–30% reductions in plasma TG (32, 36, 37). This benefit has also been observed in individuals with persistent hypertriglyceridemia even after starting the statin treatment (38) and in those with severe hypertriglyceridemia, where the reduction can reach 45% (39). Our results show an 18% reduction in TG in the ω-3 supplemented group, although similar to that observed in the ω-6 group.

Considering that cholesterol content in HDL is influenced by factors intrinsic and extrinsic to the particle, we also evaluated the effect of ω-3 in the CETP activity. CETP acts in composition and, consequently, in the structure and functionality of HDL. Abbey et al. (40) described a decrease in CETP activity in 33 hypercholesterolemic men after supplementation with 3.8 g of EPA + DHA (40), however, Calabresi et al. (41) observed no changes in the CETP concentration after supplementation with 3.6 g of EPA + DHA for 8 weeks (41). On the other hand, Raposo et al. (20) observed increased expression, concentration and activity of CETP in normolipidemic mice transgenic for CETP treated with fish oil (20). In the present study, no effects of supplementation with ω-3 rich EPA and DHA on CETP activity were observed. However, the global changes in fatty acid content represented by CP5 (cardioprotective profile) correlated negatively with the CETP activity, indicating that the intervention with ω-3 may improve the cholesterol transfer from HDL. The changes in HDL-associated NEFAs support this statement. This reduction may be a consequence of less hydrolysis of cholesterol ester bonds and TGs, leading to a reduction in free fatty acids and in the lipid transfer between lipoproteins.

The functionality of HDL is influenced by the balance with other lipoproteins and lipid content such as total cholesterol and LDL-C. Our results showed that the changes induced by both ω-3 and ω-6 fatty acids were discrete (<10%) and without differences according to the fatty acid type. The relationship between ω-3 and LDL-C is inconclusive, and some studies report increase (30, 36) and others decrease (38) in the content of cholesterol associated with this lipoprotein. Meta-analyses suggest differential effects of EPA and DHA on the LDL-C content, where DHA would be able to increase LDL-C, while EPA would lead to a reduction or a smaller increase in LDL-C (42, 43). It should be noted that, in the present study, the supplementation of ω-3 rich EPA and DHA was performed concomitantly, aiming at providing 1.11 g of EPA and 0.69 g of DHA per day. Thus, it was not possible to evaluate the differential effects of these two fatty acids.

The changes promoted by the ω-3 supplementation observed in the present study are reinforced by the monitoring done at intervention adherence, where fatty acid consumption data and the profile of incorporation of these fatty acids into HDL showed a 947% increase over usual EPA and DHA intake in study participants. The low habitual ω-3 intake observed in the present study corroborates previous studies, which classify Brazil in the category of countries with low ω-3 intake (4% of total daily caloric value) (44). The increase in ω-3 intake through the intervention was also effective in incorporating EPA and DHA into HDL particles, with an average increase of 220% in EPA content and 68% in DHA content. Augustine et al. (45) reported more than 200% increase in both fatty acids after supplementation for increase of more than 200% in both fatty acids after supplementation for 16 weeks with 3.6 g EPA + DHA, equivalent to twice the supplementation provided in our study (45). Cross-sectional studies have described that the percentage of EPA and DHA in HDL varied from 0.49 to 0.80% for EPA and 0.93 to 4.50% for DHA, depending on the study population (46, 47). The values of 0.74% of EPA and 1.96% of DHA at baseline observed in this study fall within this range.

Considering that the concept of HDL functionality includes the evaluation of the role of other HDL components, some studies have investigated the role of ω-3 in other components of this lipoprotein. Burillo et al. (19), in a clinical study, found that subjects without previous cardiovascular event, but smokers, supplemented with 1.8 g of EPA + DHA did not show modifications of Apo AI concentrations, although its expression increased (19). Previously, Bonna et al. (48) described positive correlation between EPA and Apo AI variations and negative correlation between DHA and Apo AI variations (48). Similarly, we observed a negative correlation between the change in DHA in the HDL particle and the change in Apo AI over the intervention period. This correlation was confirmed by the analysis in which we saw that increase in DHA in HDL was associated with 65% lower chance of the individual showing increased Apo AI, even after adjusting for multiple confounders (age, sex, and medications), leading us to suggest that ω-3 is associated with cholesterol enrichment in HDL and not with an increase in HDL particle number.

The antioxidant role of HDL has been related to several components of this lipoprotein, especially the content of Apo AI, Lp-PLA2 and PON1. The present study observed that the variation in PON1 activity correlated negatively with the variation in DHA. Furthermore, the incorporation of EPA and DHA was associated with a 35 and 75% lower chance, respectively, to observe an increase in the activity of this antioxidant enzyme. In the circulation, PON1 is associated almost exclusively with HDL Kontush et al. (49), and similarly to CETP, it is more present in the small, dense particles of this lipoprotein (27, 50). A previous study showed that women with rheumatoid arthritis who received 1 g of fish oil for 3 months showed a significant increase in PON1 activity (51). More recently, Golzari et al. (52) also described increased PON1 activity in individuals with DM2 after supplementation for 8 weeks with 2 g of EPA (52). However, Tanaka et al. (53) observed no modification in PON1 activity after supplementation of 1.8 g of EPA for 4 weeks in the Japanese subjects (53). Reinforcing the impact of the ω-3 intervention, the pooled fatty acid analysis performed in the present study showed that PON1 correlated positively with CP2 (cardioprotective profile). Thus, the results obtained indicate that the incorporation of ω-3 in HDL promoted the change in balance of all fatty acids in this lipoprotein, inducing the modulation of its antioxidant role. Additionally, Apo AI contributes to antioxidant capacity of HDL by redox inactivation of lipid hydroperoxides and improving transfer of lysophospholipids to the liver (54). Furthermore, recent studies showed that the arylesterase (PON-A) and lactonase (PON-L) activity affect its impact in different biological functions (55, 56). Here, we evaluated the total PON1 activity and verified that ω-3 intervention was not able to change this enzyme.

In the present study, the antioxidant capacity of HDL was also evaluated using the Lag time system. According to Kontush et al. (57) proteins and enzymes that possess antioxidant activity are non-uniformly distributed in HDL subfractions, although the highest concentrations are associated with the smallest HDL particles (57). Furthermore, it has been described that small, dense HDL particles incorporate oxidized lipids more efficiently than larger subfractions (35). The data obtained in this study show no effect of the ω-3 supplementation on increasing oxidation resistance time (lag time). However, in the ω-6 group, a slight increase in lag time (3.6%), Tmax (1.9%) was observed, as well a decrease in total content of conjugated dienes generated (+1.8%). Conversely, Park et al. (58), in an *in vitro* study using reconstituted HDL, demonstrated that particles containing ω-6 exhibited lower antioxidant power (58). Here, we also observed that a reduction in the ω-6/ω-3 ratio was associated with a 60% lower chance of an increase in lag time, signaling the interdependence of both fatty acids in HDL functionality. This relationship was confirmed when the changes in HDL-incorporated fatty acids were assessed by pooled analysis (PCA). This analysis showed that HDL particles with a more atherogenic profile (PC3) correlated positively with the generation of conjugated dienes estimated by the area under the curve obtained in Lag time analysis. Recently, Sherrat et al. (59), when assessing HDL particle oxidation upon induction by copper and in the absence of LDL, observed greater power of EPA, relative to DHA, in delaying the onset of oxidation (59). In humans, Wurm et al. (60) showed that after 12 weeks, the antioxidant function of HDL, as measured by the HDL inflammatory index, was significantly impaired in the group supplemented with ω-3 in a dose-dependent manner (60).

## Conclusion

Omega-3 supplementation over 8 weeks promoted a significant incorporation of EPA and DHA in HDL, influencing positively large HDL particles and reducing small HDL. Additionally, the incorporation of EPA and DHA in HDL were also associated with a lower chance that individuals with high cardiovascular risk had higher NEFAs, higher PON1 activity, and Apo AI. The positive effect promoted by ω-3 was modulated by ω-6/ω-3 ratio and pooled fatty acids. Based on these results, we recommend to evaluate the effect of ω-3 in other population when habitual intake of these fatty acids is present, such as Oriental population, and to stimulate individuals with high cardiovascular risk to improve their diet, especially regarding ω-3-rich foods.

## Data Availability Statement

The original contributions presented in the study are included in the article/Supplementary Materials, further inquiries can be directed to the corresponding author.

## Ethics Statement

The studies involving human participants were reviewed and approved by Ethics Committee in Research of the University Hospital (n° 1126/11) and the School of Public Health, University of São Paulo (no 2264). This study followed the Declaration of Helsinki. The patients/participants provided their written informed consent to participate in this study.

## Author Contributions

FDCC participated in the data collection, biochemistry analysis, and written manuscript. GDD contributed to the biochemistry analysis. SM reviewed critically the fatty acids analyses. NRTD was responsible for the study design and critical review of manuscript. All authors agree to be fully responsible for ensuring the completeness and accuracy of the work and to read and approve the final manuscript.

## Funding

This study was funded by the Fundação de Amparo à Pesquisa do Estado de São Paulo (FAPESP): FAPESP 2016/24531-3 (NRTD); 2011/12523-2 (NRTD); 2015/06222-0 (GDD). FDCC received scholarships from Coordenação de Aperfeiçoamento de Pessoal de Nível Superior (CAPES, Brazil).

## Conflict of Interest

The authors declare that the research was conducted in the absence of any commercial or financial relationships that could be construed as a potential conflict of interest.

## Publisher's Note

All claims expressed in this article are solely those of the authors and do not necessarily represent those of their affiliated organizations, or those of the publisher, the editors and the reviewers. Any product that may be evaluated in this article, or claim that may be made by its manufacturer, is not guaranteed or endorsed by the publisher.
