# Supplementary material for: Omega-3 Fatty Acids Improve Functionality of High-Density Lipoprotein in Individuals With High Cardiovascular Risk: A Randomized, Parallel, Controlled and Double-Blind Clinical Trial
Source: Front Nutr. 2022 Feb 23;8:767535. doi: 10.3389/fnut.2021.767535 (PMC8905646; doi:10.3389/fnut.2021.767535)
Supplement: Supplementary file 1 [file Data_Sheet_1.pdf]

**Supplements**

**TABLE S1.** HDL subfractions, by time and intervention[1]

| | **ω-6 (n=70)** | | | **ω-3 (n=77)** | | | ***p***** |
|---|---|---|---|---|---|---|---|
| **Variables** | **T0** | **T8** | **Δ%** | **T0** | **T8** | **Δ%** | |
| Percentual (%) | | | | | | | |
| HDL 1 | 10.5 (3.9) | 9.2 (3.0)* | -5.0 | 10.3 (4.0) | 11.9 (3.2)* | 28.7 | **<0.001** |
| HDL 2 | 11.5 (4.2) | 10.8 (3.5)* | -2.1 | 12.5 (4.4) | 14.2 (4.6)* | 19.3 | **<0.001** |
| HDL 3 | 6.9 (2.1) | 6.7 (2.1) | -1.2 | 7.2 (2.3) | 8.2 (2.2)* | 19.8 | **<0.001** |
| HDL 4 | 8.9 (1.7) | 8.6 (1.4) | -2.2 | 9.3 (1.8) | 9.8 (1.2)* | 8.3 | **0.001** |
| HDL 5 | 10.9 (1.8) | 10.5 (1.3) | -1.6 | 11.1 (1.6) | 11.1 (1.4) | 1.5 | 0.238 |
| HDL 6 | 21.7 (2.8) | 22.2 (2.5) | 2.8 | 21.7 (3.4) | 20.6 (3.1)* | -3.6 | **0.010** |
| HDL 7 | 7.8 (1.5) | 8.3 (1.2)* | 8.4 | 7.6 (1.7) | 7.0 (1.4)* | -2.4 | **<0.001** |
| HDL 8 | 7.7 (1.9) | 8.3 (1.6)* | 13.0 | 7.6 (2.1) | 6.8 (1.8)* | -2.5 | **<0.001** |
| HDL 9 | 6.5 (1.8) | 6.9 (1.7)* | 7.8 | 6.1 (2.0) | 5.4 (1.6)* | -6.7 | **<0.001** |
| HDL 10 | 7.6 (4.1) | 8.6 (3.8)* | 26.9 | 6.6 (4.7) | 4.9 (3.1)* | -10.6 | **<0.001** |
| Concentration (mg/dL) | | | | | | | |
| HDL-C 1 | 3.7 (2.1) | 3.6 (1.9) | 9.1 | 4.0 (1.9) | 5.0 (2.2)* | 42.9 | **0.001** |
| HDL-C 2 | 4.2 (2.4) | 4.6 (2.5)* | 11.3 | 4.8 (2.5) | 6.2 (3.1)* | 41.1 | **0.002** |
| HDL-C 3 | 2.5 (1.4) | 2.9 (1.7)* | 21.5 | 2.8 (1.5) | 3.6 (1.7)* | 41.7 | **0.029** |
| HDL-C 4 | 3.2 (1.2) | 3.4 (1.6)* | 10.4 | 3.5 (1.3) | 4.1 (1.3)* | 25.5 | **0.023** |
| HDL-C 5 | 3.8 (1.2) | 4.2 (1.2)* | 13.3 | 4.1 (1.0) | 4.5 (1.1)* | 14.0 | 0.921 |
| HDL-C 6 | 7.5 (1.8) | 8.7 (1.9)* | 18.7 | 8.1 (2.2) | 8.4 (1.9) | 8.8 | **0.001** |
| HDL-C 7 | 2.7 (0.8) | 3.3 (0.8)* | 29.6 | 2.8 (1.1) | 2.9 (0.8) | 16.5 | **0.001** |
| HDL-C 8 | 2.7 (0.9) | 3.2 (0.8)* | 33.1 | 2.8 (1.1) | 2.7 (0.8) | 9.1 | **<0.001** |
| HDL-C 9 | 2.2 (0.8) | 2.7 (0.8)* | 29.0 | 2.3 (1.0) | 2.1 (0.7) | -1.7 | **<0.001** |
| HDL-C 10 | 2.6 (1.6) | 3.4 (1.7)* | 52.3 | 2.5 (1.9) | 2.0 (1.1)* | -0.3 | **<0.001** |

[1]Values are mean (SD). *p<0.05 versus T0, p values were obtained using paired *t* or Wilcoxon tests. **p<0.05 between Δ%, p values were obtained using paired *t*-Student or Mann-Whitney tests. HDL, high density lipoprotein; HDL-C, high density lipoprotein cholesterol; Δ%, [(T8-T0)/T0]*100. P values in bold show significant differences (p < 0.05).

**FIGURE S1.** HDL antioxidant capacity, by time and intervention[1]

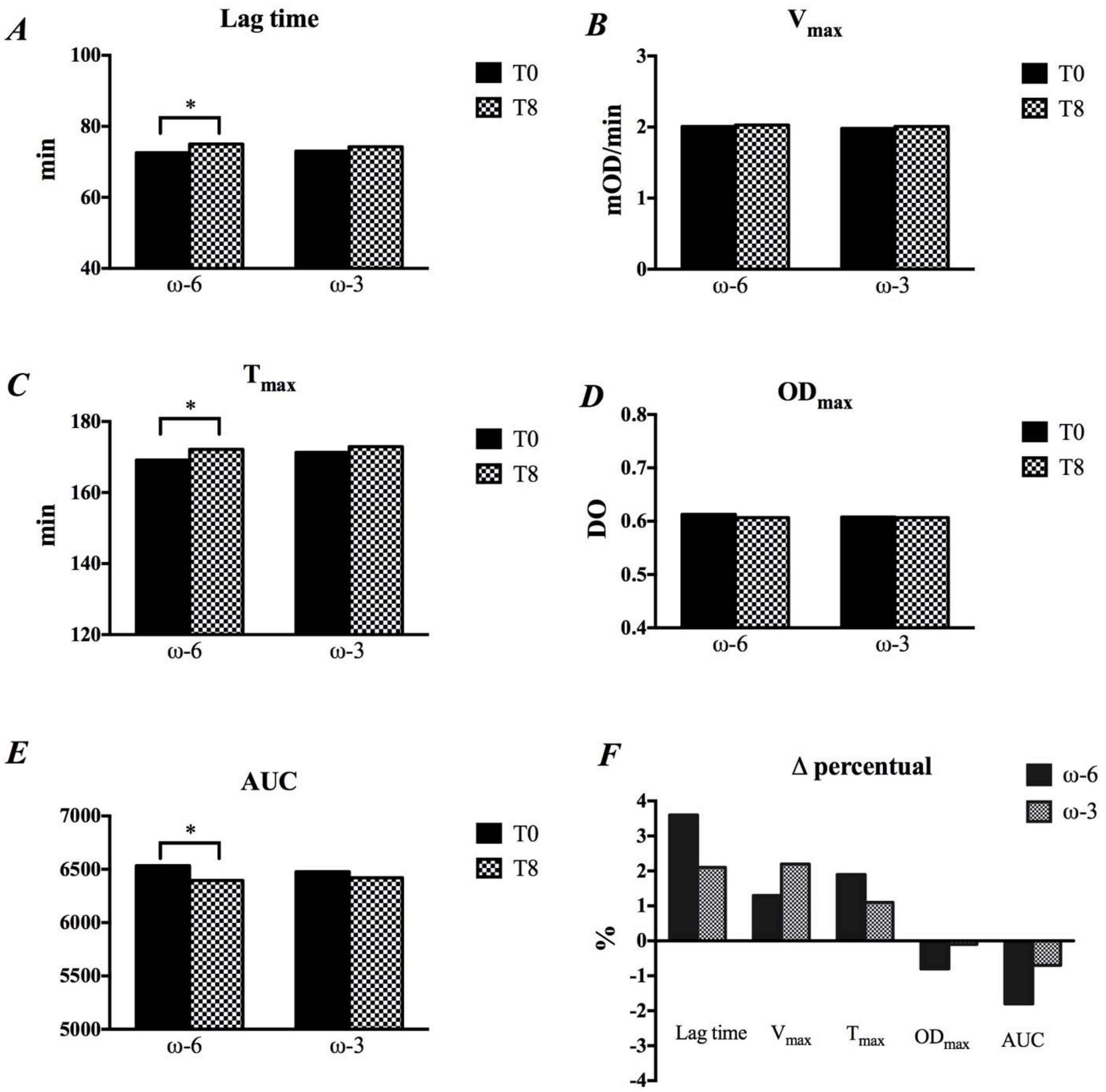


[1]*$p<0.05$, p values were obtained using paired *t*-Student or Wilcoxon tests. A) Lag time, time of oxidation resistance; B) $V_{max}$, maximum rate of lipid peroxidation; C) $T_{max}$, time for maximum production of conjugated dienes; D) $OD_{max}$, maximum production of conjugated dienes; E) AUC, area under curve; F) Δ, delta T8-T0.

**TABLE S2.** Principal Component Analysis (PCA): modifications of fatty acids in HDL[1]

| | COMPONENTS | | | | | | |
|---|---|---|---|---|---|---|---|
| **Δ FA (%)** | **PC1** | **PC2** | **PC3** | **PC4** | **PC5** | **PC6** | **PC7** |
| Δ *Myristic acid* (C14:0) | **0.273** | **0.406** | **0.638** | 0.008 | 0.183 | -0.209 | -0.393 |
| Δ *Pentadecylic acid* (C15:0) | **0.676** | -0.161 | -0.164 | -0.584 | 0.126 | -0.041 | -0.030 |
| Δ *Palmitic acid* (C16:0) | -0.521 | **0.242** | **0.589** | -0.162 | **0.250** | 0.094 | 0.191 |
| Δ *Margaric acid* (C17:0) | **0.673** | **0.435** | 0.097 | **0.207** | 0.146 | -0.364 | 0.029 |
| Δ *Arachidic acid* (C20:0) | **0.611** | -0.106 | -0.347 | -0.519 | 0.184 | -0.139 | **0.320** |
| Δ *Behenic acid* (C22:0) | **0.535** | -0.003 | -0.216 | **0.307** | -0.162 | **0.285** | -0.095 |
| Δ *Lignoceric acid* (C24:0) | **0.399** | -0.337 | 0.178 | **0.474** | -0.240 | 0.045 | **0.312** |
| Δ *Arachidonic acid* (C20:4n-6) | -0.045 | **0.407** | -0.567 | 0.182 | -0.387 | 0.019 | 0.120 |
| Δ *Stearic acid* (C18:0) | -0.027 | **0.613** | -0.089 | **0.344** | **0.337** | **0.407** | 0.145 |
| Δ *Myristoleic acid* (C14:1n-5) | **0.752** | -0.095 | -0.119 | -0.380 | 0.128 | 0.156 | -0.200 |
| Δ *10-Pentadecenoic acid* (C15:1n-5) | **0.739** | -0.181 | 0.039 | -0.284 | 0.094 | **0.253** | -0.173 |
| Δ *10-Heptadecenoic acid* (C17:1n-7) | **0.614** | 0.026 | **0.405** | 0.172 | -0.026 | -0.304 | -0.021 |
| Δ *Oleic acid* (C18:1n-9) | -0.382 | 0.183 | **0.542** | -0.292 | -0.239 | 0.145 | **0.308** |
| Δ *11-Eicosenoic acid* (C20:1n-9) | **0.838** | -0.007 | -0.028 | 0.009 | 0.054 | -0.255 | 0.196 |
| Δ *Erucic acid* (C22:1n-9) | **0.537** | -0.404 | **0.227** | 0.028 | -0.309 | **0.392** | -0.070 |
| Δ *Nervonic acid* (C24:1n-9) | **0.495** | -0.299 | 0.134 | **0.508** | -0.273 | -0.071 | **0.206** |
| Δ *α-linolenic acid* (C18:3n-3) | **0.743** | 0.063 | 0.165 | 0.098 | 0.074 | -0.400 | 0.064 |
| Δ *Eicosatrienoic acid* (C20:3n-3) | **0.446** | -0.311 | **0.238** | -0.156 | -0.282 | **0.321** | -0.094 |
| Δ *Eicosapentaenoic acid* (C20:5n-3) | -0.016 | -0.633 | 0.020 | **0.256** | **0.641** | 0.081 | -0.108 |
| Δ *Docosahexaenoic acid* (C22:6n-3) | -0.031 | -0.586 | -0.186 | **0.482** | **0.544** | 0.028 | -0.055 |
| Δ *Linoleic acid* (C18:2n-6) | -0.432 | -0.168 | -0.446 | 0.017 | -0.371 | -0.483 | -0.348 |
| Δ *γ-linolenic acid* (C18:3n-6) | **0.684** | **0.556** | 0.057 | 0.196 | 0.080 | 0.054 | -0.152 |
| Δ *Eicosadienoic acid* (C20:2n-6) | **0.657** | -0.134 | 0.188 | 0.053 | -0.407 | 0.050 | -0.113 |
| Δ *Dihomo-γ-linolenic acid* (C20:3n-6) | **0.368** | **0.656** | -0.269 | 0.161 | 0.010 | **0.318** | -0.285 |
| Δ *Docosadienoic acid* (C22:2n-6) | **0.659** | **0.234** | -0.350 | -0.076 | 0.181 | 0.064 | **0.382** |
| % variance | 15.04 | 13.19 | 12.45 | 10.35 | 9.42 | 9.14 | 8.59 |
| % accumulated variance | 15.04 | 28.23 | 40.67 | 51.02 | 60.44 | 69.57 | 78.16 |

[1]Variables displayed in loading. Method of extraction of the principal components: Eigenvalues. Kaiser criterion for sample adequacy measure = 0.657. FA, fatty acids; PC, principal components; Δ, T8-T0. The cut-off points adopted were positive values higher than 0.2.
